# Radiation precautions for inpatient and outpatient ^177^Lu-DOTATATE peptide receptor radionuclide therapy of neuroendocrine tumours

**DOI:** 10.1186/s40658-019-0243-1

**Published:** 2019-04-25

**Authors:** D. Levart, E. Kalogianni, B. Corcoran, N. Mulholland, G. Vivian

**Affiliations:** Department of Nuclear Medicine, King’s College Hospital NHS Foundation Trust, Denmark Hill, London, SE5 9RS UK

## Abstract

**Background:**

^177^Lu-DOTATATE peptide receptor radionuclide therapy is administered to patients on an inpatient and outpatient basis for the treatment of well-differentiated, metastatic neuroendocrine tumours. Following administration, these patients present an external radiation hazard due to the gamma emissions of lutetium-177. The purpose of this study was to determine precautions to be observed by ^177^Lu-DOTATATE patients to restrict the dose received by patients’ family members to less than 5 mSv in 5 years and members of the public to less than 1 mSv per year in line with the current UK legislation. Retrospective data from therapeutic administrations of ^177^Lu-DOTATATE (Mallinckrodt Pharmaceuticals) and Lutathera® (Advanced Accelerator Applications) were analysed to measure activity retention at discharge. Patient dose rate measurements were assumed to follow the same activity decay curve as that derived from a least squares fit of geometric mean counts in planar whole-body scans performed at four time points post-administration. Combining this with social contact times, the cumulative dose received through contact with the patient was estimated and an iterative process used to determine the length of contact restrictions to ensure the relevant dose constraints are not exceeded.

**Results:**

On average, 36% of the administered activity was retained at the time of discharge for inpatients receiving ^177^Lu-DOTATATE (Mallinckrodt). Retentions of 24% and 38% were measured for Lutathera® inpatients and outpatients respectively. Inpatients should restrict day contact and sleep separately from their partner for 15 days and remain off work for 5 days post-therapy. Contact with children for whom the patient is the main carer should be restricted for 16, 13 and 9 days for children below 2, 2–5 and 5–11 years respectively. One additional day is added to outpatient restriction periods, except for children aged 2–5 years which remains 13 days. No private transport restrictions are required. Patients should limit travel by public transport to 1 h on the day of discharge.

**Conclusion:**

Restrictions are necessary to limit radiation dose to members of patients’ household and the public. Proposed precautions for inpatient and outpatient ^177^Lu-DOTATATE therapy protocols restrict the dose received to less than the limit imposed by the UK legislation.

## Background

Lutetium-177 labelled somatostatin analogues such as ^177^Lu-DOTATATE are administered for peptide receptor radionuclide therapy (PRRT) of well-differentiated, metastatic neuroendocrine tumours (NETs). The ^177^Lu-DOTATATE binds to somatostatin receptors that are overexpressed on the surface of NET cells, thereby providing a means of delivering targeted radiation directly to the tumour. ^177^Lu-DOTATATE has been shown to improve tumour response rates and progression-free survival compared with alternative treatments whilst resulting in few serious adverse effects [1, 2], which is consistent with the outcomes of Lutathera® treatment in the randomised phase 3 Neuroendocrine Tumours Therapy (NETTER-1) trial [3].

Patients will typically undergo four therapy cycles each consisting of 7.4 GBq ^177^Lu-DOTATATE at 6–12-week intervals [4]. ^177^Lu has a physical half-life of 6.73 days and emits β particles with maximum energy 0.498 MeV, relating to a maximum soft-tissue penetration depth of 1.7 mm [4]. In addition to the β particles, ^177^Lu has two gamma emissions at 113 keV and 208 keV with low relative abundance (6.2% and 10.4% respectively) [5]. Whilst proving advantageous as a means of post-therapy imaging and dosimetry measurements, these γ emissions present an external radiation hazard to those who come into contact with the patient, particularly hospital staff, family and members of the public. Another source of risk arises from the biological excretion of the radiopharmaceutical that occurs primarily through the urine and the majority of which takes place in the first 2 days following treatment [4, 6]. For these reasons, many centres necessitate inpatient isolation after ^177^Lu-DOTATATE administration with precautions to be followed once the patient leaves the hospital in order to limit the radiation dose to family members and the public. Local regulatory requirements dictate the length of patient confinement and some countries allow for ^177^Lu-DOTATATE therapy on an outpatient basis. Work by Calais and Turner [7] has shown that outpatient therapy is acceptable within Australia’s regulatory framework where this is routinely carried out, and Olmstead et al. have demonstrated the safety and feasibility of an outpatient protocol in Canada [8].

The UK legislative implementation of the new European Commission Basic Safety Standards (BSS) (Ionising Radiations Regulations 2017 (IRR17) [9]) is based on recommendations from the International Commission on Radiological Protection (ICRP) and specifies that the effective dose limit to a member of public should not exceed 1 mSv per year [10, 11]. The legislation has the provision that the dose limit may be averaged over 5 years (5 mSv in 5 years) to allow for doses greater than 1 mSv in any single year to friends and family members who may be in close proximity to the patient but cannot be treated as a comforter and carer [9]. Working within these regulations, ^131^I-NaI has been safely administered for hyperthyroidism on an outpatient basis for many years with well-established restriction times [12, 13]. The gamma emission energies (relative abundance) of ^131^I are 284 keV (6.1%), 364 keV (81.2%) and 642 keV (7.3%). With comparatively lower γ emission energies and relative abundance as well as fewer routes of biological excretion than ^131^I, the properties of ^177^Lu are favourable in terms of minimising public radiation exposure and it is proposed that similar precaution periods should be established for ^177^Lu-DOTATATE PRRT.

^177^Lu-DOTATATE therapies have been performed at King’s College Hospital since 2007 on an inpatient basis, with outpatient therapies introduced in 2012. Inpatient ^177^Lu-DOTATATE therapies are typically carried out in the afternoon with the patient staying overnight in isolation and then discharged the following day. The outpatient protocol requires the patient to attend the Nuclear Medicine Department at 08:30 to begin preparation for ^177^Lu-DOTATATE administration, which should be commenced by 09:00 to maximise the length of time the patient spends in the department before being discharged at 17:00. Prior to 2013, generic ^177^Lu-DOTATATE was supplied by Mallinckrodt Pharmaceuticals (Mallinckrodt plc, Staines-upon-Thames, UK) and intravenously administered in approximately 90 ml at an infusion rate of 250 ml/h. Thereafter, the ^177^Lu-DOTATATE (Lutathera®) was supplied by Advanced Accelerator Applications (AAA, Saint-Genis-Pouilly, France) and administered in 23–25 ml at an infusion rate of 60 ml/h. For renal protection, patients have a co-infusion of 50 g amino acids (25 g Lysine/25 g Arginine) (Preston Pharmaceuticals, Preston, UK) diluted in 1 l of normal saline starting 30 min prior to the ^177^Lu-DOTATATE administration and maintained for 4 h. All patients undergo a glomerular filtration rate (GFR) measurement prior to each therapy cycle using the ^51^Cr-EDTA plasma clearance method [14] to assess renal function.

The purpose of this study was to calculate the percentage retained activity at discharge for inpatient and outpatient therapies, estimate the equivalent dose received by those in contact with the patient after discharge and determine appropriate radiation precautions to be followed by ^177^Lu-DOTATATE therapy patients in order to comply with the UK legislation.

## Method

### ^177^Lu-DOTATATE retention and dose rate measurements

Retrospective post-^177^Lu-DOTATATE PRRT imaging and dose rate data, acquired as part of the standard departmental therapy protocol, were analysed to measure ^177^Lu-DOTATATE retention at discharge and establish appropriate radiation protection precautions. This data included 32 inpatient Mallinckrodt ^177^Lu-DOTATATE administrations (16 patients) collected from 2007 to 2011, as well as 20 outpatient Lutathera® administrations (15 patients) and 24 inpatient Lutathera® administrations (18 patients) collected from 2013 to 2014. The imaging data comprised serial planar whole-body scans using either a Siemens Symbia T16 gamma camera (Siemens Healthineers, Erlangen, Germany) with MELP collimators or Philips Skylight (Philips Healthcare, Eindhoven, Netherlands) with MEGP collimators. Mallinckrodt ^177^Lu-DOTATATE patients underwent post-therapy imaging at four time points; immediately following infusion and before voiding (*T*_0_), prior to discharge (*T*_D_) at approximately *T*_0_ + 24 h, 4/5 days and 6/7 days post-administration. Lutathera® patients underwent whole body imaging at *T*_0_ and *T*_D_, which occurred at approximately *T*_0_ + 8 h for outpatients and *T*_0_ + 24 h for inpatients. Regions of interest (ROIs) over the anterior and posterior whole body scans were drawn manually using the HERMES Hybrid Viewer™ (Hermes Medical Solutions, Stockholm, Sweden) application. The geometric mean of the total counts in the anterior and posterior views at *T*_0_ and *T*_D_ were used to determine the percentage ^177^Lu retention at discharge. Dose rate measurements were taken at 0.1 and 1 m from the right lateral mid-abdomen of all Mallinckrodt ^177^Lu-DOTATATE patients at *T*_0_ using a G-M probe (Series 900 mini-monitor probe type D). The dose rate at 1 m from the anterior mid-abdomen was recorded for 13 outpatient Lutathera® administrations (11 patients) at *T*_D_.

### Dose estimation and radiation protection precautions

To estimate the equivalent dose received by those who come into contact with ^177^Lu-DOTATATE PRRT patients and establish appropriate radiation protection precautions, the Mallinckrodt ^177^Lu-DOTATATE imaging and dose rate data were analysed alongside estimated patterns of close contact and compared against the UK legislation. Using ROI analysis, the geometric mean counts from anterior and posterior whole body images were plotted over time and regression analysis using a least squares method was used to derive activity retention curves. With *T*_0_ images acquired prior to the patient emptying their bladder, the whole-body image counts at this time point represent 100% of the activity administered. The retention of ^177^Lu-DOTATATE within the body as a percentage of total administered activity was modelled as a bi-exponential decay process, where the activity (*A*_*t*_) at time (*t*) after administration of initial activity (*A*_*0*_) is described by the following equation:

$$ {A}_t={A}_0\left({A}_s{e}^{-{\lambda}_st}+{A}_l{e}^{-{\lambda}_lt}\right) $$

*A*_*s*_ and *A*_*l*_ are the constants of the short and long component respectively and *λ*_*s*_ and *λ*_*l*_ are the effective decay constants of the short and long component, with effective half-lives *T*_*s*_ and *T*_*l*_ respectively. It was assumed that the *T*_0_ external dose rate measured at 0.1 and 1 m decayed according to the decrease in retention of the radiopharmaceutical within the patient. To estimate the dose received by the patients’ family members and members of the public once the patient leaves the hospital, published data on social contact times were applied using the Barrington et al. method [15] which made the following assumptions:

1. The patient will spend 6 h per day at 1 m, followed by 8 h sleeping at 0.1 m from their partner.
2. Assuming that the patient is the parent or primary carer of a child the following contact patterns, depending on the age of the child, were applied:
  - For a young infant of less than 2 years, the patient will spend 15 periods of 35 min per day at 0.1 m from the child.
  - For a child between 2 and 5 years, the patient will spend 4 h at 0.1 m and 8 h at 1 m per day from the child.
  - For a child between 5 and 11 years, the patient will spend 2 h at 0.1 m and 4 h at 1 m per day from the child.
3. The patient will spend an 8 h working day at 1 m from the same colleague on return to work.
4. The patient will sit 1 m from the driver with no other passengers present for travel by private transport and 0.1 m from fellow passengers for travel by public transport.

The equivalent dose to individuals within each category defined above was calculated using the dose rate decay curve by summing the dose received during the corresponding contact period. The contact was assumed to start immediately after the patient leaves the hospital and at the beginning of each subsequent 24 h period. Any dose received after 20 days or at a distance greater than 1 m was assumed to be negligible. Restriction times were calculated by iteratively finding the number of whole days post-discharge to limit contact so that the relevant dose constraint is not exceeded. For outpatient therapies, the length of precautions was determined by extrapolating the inpatient dose rate decay curve back to the expected outpatient time of discharge (*T*_0_ + 8 h) and applying the same iterative process. Under the standard protocol for ^177^Lu-DOTATATE therapy, it is likely that patients will undergo four treatment cycles in 1 year. Therefore, applying the 5 mSv in 5 years’ provision for the patients’ close friends and family members a 1 mSv dose constraint for each cycle was applied to calculate appropriate restriction periods to comply with the UK legislation ensuring that no dose limit is exceeded. The 1 mSv per year dose limit is applied to patients’ work colleagues and other passengers when travelling, with a 0.3 mSv constraint imposed per treatment cycle.

## Results

### ^177^Lu-DOTATATE retention and dose rate measurements

The mean administered activity for inpatient Mallinckrodt ^177^Lu-DOTATATE therapy administrations was 7079 MBq (range 5925–7890 MBq) and for Lutathera® administrations was 7577 MBq (range 7076–8038). The mean ^177^Lu retention at discharge and length of stay in the hospital for outpatient and inpatient therapies with ^177^Lu-DOTATATE from both suppliers are shown in Table 1. The mean retained activity for inpatients receiving Lutathera® was found to be 33% lower compared with Mallinckrodt ^177^Lu-DOTATATE. On average, patients receiving Lutathera® as an outpatient will retain an additional 1091 MBq at *T*_D_ compared with those staying as an inpatient.

**Table 1:** Mean (SD) [range] administered activity, length of stay in hospital, percentage ^177^Lu and activity retention at discharge for outpatient and inpatient administrations

	Number of administrations	Number of patients	Administered activity (MBq)	Time *T*_0_–*T*_D_ (h)	^177^Lu retention (%)	Retained activity (MBq)
Inpatients	32	16	7079 (419) [5925–7890]	18.1 (1.5) [16.2–21.2]	36 (15) [15–62]	2537 (1079) [1087–4482]
^177^Lu-DOTATATE Mallinckrodt						
Inpatients	24	18	7545 (204) [7076–7913]	18.2 (1.1) [16.7–21.0]	24 (7) [13–37]	1806 (494) [984–2740]
Lutathera® AAA						
Outpatients	20	15	7608 (242) [7259–8038]	5.2 (0.7) [3.6–6.3]	38 (8) [28–58]	2897 (663) [2103–4670]
Lutathera® AAA						

The mean dose rates at 0.1 m and 1 m from the right lateral mid-abdomen of the patient immediately after Mallinckrodt ^177^Lu-DOTATATE administration were 407 μSv/h (range 250–750 μSv/h) and 20 μSv/h (range 15–25 μSv/h) respectively. Dose rate data were only available for 13 outpatient Lutathera® administrations (11 patients) who received a mean activity of 7700 MBq (range 7265–8038 MBq). The mean dose rate at 1 m from the anterior mid-abdomen was 15 μSv/h (range 5–25 μSv/h) at *T*_D_, which had a mean of 5.7 h (range 3.5–6.9 h) post-administration.

### Activity decay curves

**Fig. 1:**
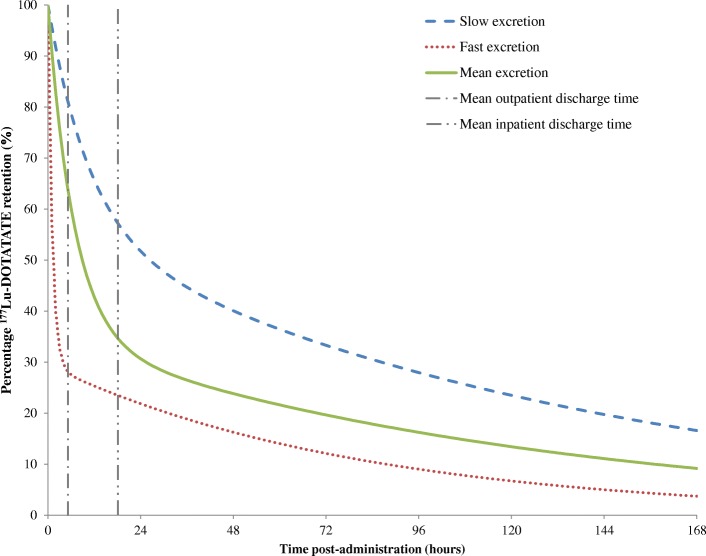
Examples of fitted ^177^Lu-DOTATATE retention curves up to 168 h post-administration for patients with slow (*A*_*s*_ = 0.44, *A*_*l*_ = 0.56, *T*_*s*_ = 7.4, *T*_*l*_ = 95.6) and fast excretion (*A*_*s*_ = 0.76, *A*_*l*_ = 0.24, *T*_*s*_ = 3.9, *T*_*l*_ = 67.1). The mean curve fit and times of outpatient and inpatient discharge are also shown

The mean time after *T*_0_ for subsequent imaging events is given in Table 2 along with the mean percentage ^177^Lu-DOTATATE retention measured at each time point. The mean half-life (± 1 SD) of the short and long decay components of the bi-exponential fit to the inpatient Mallinckrodt ^177^Lu-DOTATATE activity decay curves were 4.7 (± 1.4) hours and 87.2 (± 14.7) hours respectively and the mean constants *A*_*s*_ and *A*_*l*_ were 0.65 (± 0.17) and 0.35 (± 0.17). A good fit was achieved for all sets of data using the bi-exponential least square fit with a minimum correlation coefficient of 0.984. Examples of ^177^Lu-DOTATATE fitted retention curves for patients with fast and slow excretion are shown in Fig. 1 along with the mean curve fit.

**Table 2:** Mean (SD) [range] post-therapy scan times with corresponding percentage ^177^Lu retention for inpatient administrations

Approximate post-therapy scan time (hours/days)	Number of administrations	Post-therapy scan time (hours)	^177^Lu retention (%)
24/D1/*T*_D_	32	18.1 (1.5) [16.2–21.2]	33 (14) [15–62]
96/D4	30	93.3 (1.8) [88.2–97.2]	17 (10) [7–37]
120/D5	8	114.6 (2.2) [111.3–118.1]	14 (6) [7–24]
144/D6	2	139.0 (2.0) [137.6–140.4]	10 (6) [6–15]
168/D7	24	164.0 (2.2) [161.1–170.0]	10 (7) [4–24]

Assuming that patients had undergone an outpatient therapy, the mean predicted retention at *T*_D_ estimated from the Mallinckrodt ^177^Lu-DOTATATE activity decay curves was 63% (range 40–80%). The estimated dose rates and dose rate per MBq administered activity at *T*_D_ for inpatients and outpatients extrapolated from the dose rate decay curves are given in Table 3 alongside measured values at *T*_0_ and *T*_D_.

**Table 3:** Mean (SD) [range] measured and estimated dose rates and dose rate per MBq administered activity at 0.1 and 1 m from the mid-abdomen

	Time (hours)	Dose rate (μSv/h)		Dose rate per MBq (nSv/h/MBq)	
		0.1 m	1 m	0.1 m	1 m
^177^Lu-DOTATATE Mallinckrodt Inpatient measured	0	407 (83) [250–750]	20 (2) [15–25]	58 (12) [35–99]	3 (0.3) [2.1–3.6]
^177^Lu-DOTATATE Mallinckrodt Inpatient estimated	18.1	148 (77) [49–334]	7 (3) [4–15]	21 (11) [5–46]	1 (0.5) [0.4–2]
^177^Lu-DOTATATE Mallinckrodt Outpatient estimated	5.2	258 (75) [100–435]	13 (3) [6–20]	37 (10) [14–60]	2 (0.4) [0.8–3]
Lutathera® AAA Outpatient measured	5.7	–	15 (5) [5–25]	–	2 (0.6) [0.6–3]

### Dose estimation and radiation protection precautions

If no contact restrictions were put in place, the cumulative dose per treatment cycle received by family members and the public from patients undergoing ^177^Lu-DOTATATE therapy are given in Table 4. With the exception of private transport, these dose estimates approach or exceed the established constraints for all groups and demonstrate that precautions are necessary when ^177^Lu-DOTATATE therapy patients leave the hospital. The total dose to a driver for an 8-h journey by private transport immediately after leaving the hospital is below the 0.3 mSv/year dose constraint for members of the public for both inpatients and outpatients. Therefore, there are no restrictions required for journeys by private transport following ^177^Lu-DOTATATE therapy.

**Table 4:** Mean (SD) [range] equivalent dose (mSv) per cycle to close relatives and members of the public for inpatient and outpatient ^**177**^Lu-DOTATATE administrations resulting from no restriction on contact with patient

	Partner	Child < 2 years	Child 2–5 years	Child 5–11 years	Work colleague	8 h private transport	2 h public transport
Inpatients	6.2 (4.6) [2.0–17.9]	7.0 (5.2) [2.3–20.4]	3.5 (2.5) [1.2–9.8]	1.7 (1.3) [0.6–4.9]	0.3 (0.2) [0.1–0.8]	0.05 (0.03) [0.02–0.1]	0.3 (0.2) [0.1–0.7]
Outpatients	7.1 (5.1) [2.4–20.0]	8.2 (5.7) [2.6–22.8]	4.0 (2.7) [1.4–11.0]	2.1 (1.4) [0.7–5.5]	0.3 (0.2) [0.2–0.8]	0.07 (0.03) [0.03–0.1]	0.4 (0.2) [0.1–0.8]

The mean length of contact restrictions, starting when the patient leaves the hospital and prohibiting any contact with the patient at a distance of less than 1 m, required to comply with the UK legislation are shown in Table 5. The upper 95th percentile of the restriction times is taken as the recommended period for radiation precautions to remain in place for each exposed group. The patient should restrict day contact and sleep separately from their partner for 15 days post-therapy for inpatient treatment protocols. If no contact restrictions were advised, the mean dose per treatment cycle received by the patient’s partner would be 6.2 mSv (range 2.0–17.9 mSv). Contact with children for whom the patient is the parent or main carer should be restricted for 16 days for children below 2 years of age, 13 days for children aged 2–5 years and 9 days for children aged 5–11 years. Without restrictions, the mean dose received by children in the respective age groups would be 7.0 mSv (range 2.3–20.4 mSv), 3.5 mSv (range 1.2–9.8 mSv) and 1.7 mSv (range 0.6–4.9 mSv). The patient should also remain off work for 5 days post-therapy; otherwise, the mean dose received by any work colleague spending an 8-h working day at 1 m from the patient would be 0.3 mSv (range 0.1–0.8 mSv) per treatment cycle.

**Table 5:** Mean (upper 95th percentile) [range] restriction durations for inpatient and outpatient ^177^Lu-DOTATATE administrations to limit dose for close relatives to 1 mSv per cycle and 0.3 mSv per year for members of the public

Restriction	Inpatient therapy period (days)	Outpatient therapy period (days)
Restrict day contact < 1 m and sleep apart from partner	8 (15) [3–15]	9 (16) [4–16]
Restrict contact < 1 m with < 2 year old child	9 (16) [4–16]	9 (17) [5–17]
Restrict contact < 1 m with child aged 2–5 years	5 (13) [1–13]	6 (13) [1–13]
Restrict contact < 1 m with child aged 5–11 years	3 (9) [0–9]	3 (10) [1–10]
Remain off work	1 (5) [0–5]	1 (6) [0–6]

For patients treated following an outpatient protocol, the patient should restrict day contact and sleep separately from their partner for 16 days post-therapy. If no contact restrictions were advised, the mean dose per treatment cycle received by the patient’s partner would be 6.0 mSv (range 2.4–20.0 mSv). Contact with children for whom the patient is the parent or main carer should be restricted for 17 days for children below 2 years of age, 13 days for children aged 2–5 years and 10 days for children aged 5–11 years. Without restrictions, the mean dose received by children in the respective age groups would be 8.2 mSv (range 2.6–22.8 mSv), 4.0 mSv (range 1.4–11.0 mSv) and 2.1 mSv (range 0.7–5.5 mSv). The patient should also remain off work for 6 days post-therapy; otherwise, the mean dose received by any work colleague spending an 8-h working day at 1 m from the patient would be 0.3 mSv (range 0.2–0.8 mSv) per treatment cycle.

Table 6 shows the number of hours of travel on public transport permitted on days 0–9 post-therapy in order to maintain the 0.3 mSv/year dose constraint for members of the public. The lower fifth percentile of the number of hours travel allowed each day is taken as the recommended period for travel by public transport. On the day the patient leaves the hospital travel by public transport should be limited to 1 h and increases to 3 h at 9 days post-therapy.

**Table 6:** Mean (lower 5% percentile) [range] number of hours (rounded to the nearest hour) travel allowed on public transport at 0–9 days post-therapy to limit dose for members of the public to 0.3 mSv per year

No. of days post-therapy	0	1	2	3	4	5	6	7	8	9
Inpatients	–	3 (1) [1–6]	4 (1) [1–8]	5 (1) [1–11]	6 (1) [1–13]	8 (1) [1–16]	10 (2) [2–21]	13 (2) [2–28]	16 (2) [2–39]	21 (3) [3–55]
Outpatients	2 (1) [1–5]	3 (1) [1–7]	4 (1) [1–9]	5 (1) [1–11]	6 (1) [1–14]	8 (1) [1–17]	11 (2) [2–23]	14 (2) [2–31]	18 (3) [3–43]	24 (3) [3–63]

## Discussion

The percentage retained activity at the time of discharge was measured retrospectively for PRRT patients who were administered ^177^Lu-DOTATATE supplied by two different companies, following both inpatient and outpatient therapy protocols. This study demonstrated a significant difference in the administered activity and percentage retention at discharge between the two ^177^Lu-DOTATATE products, a 6% higher mean administered activity and 33% lower mean retention for inpatients receiving Lutathera® compared to those treated with ^177^Lu-DOTATATE from Mallinckrodt. Similarly, the measured retention at discharge for Lutathera® outpatients was 40% lower than that predicted from the bi-exponential fit to Mallinckrodt inpatient curves at 5.2 h post-administration. The amount of activity retained will vary from one patient to another depending on the extent of disease and somatostatin avidity, radiopharmaceutical uptake and kidney function. Binding of the radiolabelled peptide to somatostatin receptors expressed by NET cells is known to be altered by the specific activity of the radiopharmaceutical [16]. Therefore, any variation in ^177^Lu-DOTATATE production between the two suppliers could also result in differences in radiopharmaceutical retention. Detailed investigation of the production methods and exact composition of the two radiopharmaceuticals was outside the scope of study and the extent to which this impacts the activity retention remains unknown. Additionally, the patients’ total tumour burden and avidity were not examined and it is therefore also unclear how this could have influenced the data.

In previous studies involving ^177^Lu-DOTATATE therapy, Kwekkeboom et al. [6] report a mean urinary excretion of 64% in the first 24 h after infusion, which is comparable to the measured 36% retained activity at discharge for Mallinckrodt ^177^Lu-DOTATATE inpatients. Calais and Turner [7] found that outpatients released on reaching a dose rate of 25 μSv/h at 1 m had excreted 46% administered activity within 4 h. This result lies between the percentage retention measured for outpatient Lutathera® administrations and that predicted by extrapolating the Mallinckrodt ^177^Lu-DOTATATE activity decay curve back to the time of outpatient discharge. A direct comparison of these studies is difficult due to the fact that both sets of authors administered ^177^Lu-DOTATATE that was synthesised locally on each site, resulting in possible differences in specific activity that may be affecting radiopharmaceutical retention as well as other factors described above.

For outpatient Lutathera® therapies, the mean retained activity at discharge is approximately 1 GBq more than that for inpatients. The additional activity will be excreted over the following hours to the equivalent level had the patient stayed overnight, which leads to an increased contamination risk for outpatient therapies as less activity is expelled whilst in a controlled hospital environment. Since ^177^Lu-DOTATATE excretion occurs primarily through the urine, the risk of contamination is reduced through good hand hygiene, double-flushing of the toilet, asking male patients to urinate sitting down and the use of a separate bathroom where available. The reduced retention of Lutathera® compared with Mallinckrodt ^177^Lu-DOTATATE may result in a lower contamination risk for this radiopharmaceutical when patients are discharged from hospital since more activity will have already been excreted.

The estimated dose to patients’ family and members of the public demonstrated that contact restrictions are necessary after discharge from hospital in order to meet regulatory requirements in the UK. Recommended precaution periods for contact with family members, work colleagues and travel by public transport have been proposed for ^177^Lu-DOTATATE therapies and are given in Table 7. An outpatient therapy protocol does not lead to significantly longer contact restrictions, and differences are a result of the additional day the patient will remain isolated in the hospital as an inpatient. The possibility of outpatient therapies allows for a higher throughput of patients since this is no longer limited by the availability of inpatient isolation suites, an associated cost saving per therapy cycle and an improved patient experience through increased convenience and removing the anxiety related to an overnight stay in hospital.

**Table 7:** Recommended restriction durations for inpatient and outpatient ^177^Lu-DOTATATE administrations to limit dose for close relatives to 1.0 mSv per cycle and 0.3 mSv per year for members of the public

Restriction	Inpatient therapy period	Outpatient therapy period
Restrict day contact < 1 m and sleep apart from partner	15 days	16 days
Restrict contact < 1 m with < 2-year-old child	16 days	17 days
Restrict contact < 1 m with child aged 2–5 years	13 days	13 days
Restrict contact < 1 m with child aged 5–11 years	9 days	10 days
Remain off work	5 days	6 days
Private transport	No restriction	No restriction
Public transport at time of discharge	1 h	1 h
Public transport at 9 days post-therapy	3 h	3 h

It should be noted that within the UK regulatory framework under the Ionising Radiation (Medical Exposure) Regulations 2017 (IR(ME)R17) [17] legislation, there exists the ability to designate persons exposed to ionising radiation in the care and support of patients as ‘carers and comforters’. Except in rare circumstances, these are typically adult friends and family members of the patient who consent to incur the radiation exposure having been fully informed of the risks involved. While dose constraints must be established, carers and comforters are not subject to the dose limits imposed by IRR17. The detriment to the carer and comforter must be weighed against the possible health and psychological benefit to the patient and/or relative or friend by the IR(ME)R practitioner whilst justifying the patient’s therapeutic exposure. The restriction durations recommended in this study are intended for use as guidance in situations where determination of precaution length on an individual patient basis is not possible and where designation as carer and comforters is not justified or practical, such that both inpatient and outpatient ^177^Lu-DOTATATE PRRT procedures can be undertaken and considered safe in accordance with IRR17.

As a result of selecting the upper 95th percentile of data, the recommended precautions are conservative and likely to be an overestimation for most patients, which is evident when compared to the mean precaution duration from the inpatient data. An additional overestimation exists when applying these restrictions to Lutathera® patients since the length of precautions were determined using Mallinckrodt ^177^Lu-DOTATATE data that has been demonstrated to result in increased activity retention at discharge compared to Lutathera® patients. This will be counteracted in part by the increased administered activity for Lutathera® patients. The dose received by an individual from a patient undergoing ^177^Lu-DOTATATE therapy will depend on the time spent in contact with the patient. The social contact intervals used in estimating doses received by family members and the public were assumed to occur at the start of each 24 h period, beginning at 8 h post-administration for outpatients and 24 h for inpatients. This will result in further overestimation of the calculated dose as it is unlikely that this will be the case in a typical home setting, with patient contact spread more evenly over each 24-h period. Despite these considerations, the proposed length of precautions is comparable with the advice given to thyroid cancer patients receiving 1850 MBq ^131^I-NaI for ablation [15], indicating that they can be considered achievable and acceptable for the majority of patients. The social contact times applied in this study are assumptions based on the limited data available in the literature and may differ dramatically from patient to patient. As such, each patient’s personal circumstances should be taken into consideration by the healthcare professional when providing radiation protection advice. Using the method described in this study, the precaution duration can be modified on an individual patient basis for those with atypical home circumstances that find the length of precautions challenging or for patients who might receive more than four therapy cycles over a 5-year period.

Universal implementation of the new European Commission Basic Safety Standards suggests that these proposals could be considered in developing ^177^Lu-DOTATATE treatment protocols for patients with metastatic NET throughout the European Union, and these results may be used to inform similar recommendations for other countries subject to locally applicable radiation safety regulations.

## Conclusion

Estimated doses to members of patients’ household and the public have shown that contact restrictions are necessary following ^177^Lu-DOTATATE therapy to comply with the UK legislation. Radiation precautions based on measured activity retention, initial dose rates and social contact times have been proposed and are considered achievable for most patients. Implementing these precautions restricts the dose received by family members to less than 5 mSv in 5 years and members of the public to less than 1 mSv per year, demonstrating that ^177^Lu-DOTATATE therapy can be administered safely on both an inpatient and outpatient basis.

## Abbreviations

AAA: Advanced Accelerator Applications
GFR: Glomerular filtration rate
IR(ME)R17: Ionising Radiation (Medical Exposure) Regulations 2017
IRR17: Ionising Radiations Regulations 2017
NET: Neuroendocrine tumour
PRRT: Peptide receptor radionuclide therapy
ROI: Regions of interest
SD: Standard deviation
